# Interval of Uncertainty: An Alternative Approach for the Determination of Decision Thresholds, with an Illustrative Application for the Prediction of Prostate Cancer

**DOI:** 10.1371/journal.pone.0166007

**Published:** 2016-11-09

**Authors:** Johannes A. Landsheer

**Affiliations:** Department of Methodology and Statistics, Utrecht University, Utrecht, the Netherlands; University of Oklahoma Health Sciences Center, UNITED STATES

## Abstract

Often, for medical decisions based on test scores, a single decision threshold is determined and the test results are dichotomized into positive and negative diagnoses. It is therefore important to identify the decision threshold with the least number of misclassifications. The proposed method uses trichotomization: it defines an Uncertain Interval around the point of intersection between the two distributions of individuals with and without the targeted disease. In this Uncertain Interval the diagnoses are intermixed and the numbers of correct and incorrect diagnoses are (almost) equal. This Uncertain Interval is considered to be a range of test scores that is inconclusive and does not warrant a decision. It is expected that defining such an interval with some precision, prevents a relatively large number of false decisions, and therefore results in an increased accuracy or correct classifications rate (CCR) for the test scores outside this Uncertain Interval. Clinical data and simulation results confirm this. The results show that the CCR is systematically higher outside the Uncertain Interval when compared to the CCR of the decision threshold based on the maximized Youden index. For strong tests with a very small overlap between the two distributions, it can be difficult to determine an Uncertain Interval. In simulations, the comparison with an existing method for test-score trichotomization, the Two-graph Receiver Operating Characteristic (TG-ROC), showed smaller differences between the two distributions for the Uncertain Interval than for TG-ROC’s Intermediate Range and consequently a more improved CCR outside the Uncertain Interval. The main conclusion is that the Uncertain Interval method offers two advantages: 1. Identification of patients for whom the test results are inconclusive; 2. A higher estimated rate of correct decisions for the remaining patients.

## Introduction

Medical decision-making is often binary in nature, such as the decision whether an illness is present or absent in a patient or whether patients should be treated or not. Applying a well-established standard to test scores results in two distributions, one for patients who require treatment according to the standard and one for patients for whom treatment is not or not yet necessary. The idea of the basic method for the determination of decision thresholds is straightforward: patients with a test score below a suitable decision threshold are considered a non-case and alternative explanations are considered or the patient is sent home, while the patients with a test score above the threshold are treated [1]. Unfortunately, test scores often show some overlap between the two distributions and therefore yield false positives (FP) and false negatives (FN). This provides a challenge for the determination of a threshold that limits these mistakes and maximizes true positives (TP) and true negatives (TN). In addition, there is a possibility of uncertainty for some patients [2,3] and the question may arise whether or not all available test scores can determine the disorder sufficiently. To facilitate this dichotomization, a methodology of decision threshold or cut-point determination has been developed.

### Dichotomization Methods

Lopez-Raton, Rodrıguez-Alvarez, Cadarso-Suárez, and Gude-Sampedro [4] present a collection of more than thirty different dichotomization methods for the determination of single decision thresholds. In all of these methods, both the proportion of positives that are correctly identified (Sensitivity: Se = TP / (TP + FN)) and the proportion of negatives that are correctly identified (Specificity: Sp = TN / (TN + FP)) play a central role. Both Se and Sp are calculated for all possible thresholds of the test to identify the threshold with the lowest number of misclassifications. Such an optimal decision threshold inevitably lies in the overlap area. Many of these methods use additional information, such as information about costs and benefits. Unfortunately, it is difficult to come to clear conclusions concerning the applicability of these decision thresholds. Cammann, Jung, Meyer, and Stephan [5] conclude there is no universally suitable model for the determination of single cut-points, but rather a set of models, each of which is suitable for a specific population. Most importantly, for every single cut-point method, there is a trade-off between sensitivity and specificity for each cut-point [6] and choosing a higher or lower cut-off exchanges one error type for another. Furthermore, maximization of one criterion (for instance sensitivity) in a sample may offer a solution for that sample, but there is no guarantee that this maximization is also valid for other samples.

In the dichotomous single threshold methodology, the Receiver Operating Characteristic (ROC) is an important tool. The ROC curve shows the True Positive Rate (TPR or Sensitivity) against the False Negative Rate (FNR = 1 –Specificity) over all possible decision threshold values of the test. The Youden index is the most frequently used for decision threshold determination, which is defined as TPR–FNR (= Se + Sp -1). The associated decision threshold defines the threshold for which TPR–FNR is maximized and is therefore considered optimal [7].

As a method, the ROC approach has several attractive properties [8]: It evaluates the discriminatory ability of a test to assign patients to two classifications, for instance a group without the targeted condition (‘healthy’) and a group with the targeted condition (‘diseased’). It allows for finding an optimal decision threshold that minimizes misclassifications. The ROC approach enables comparison between the efficacies of two or more tests. Furthermore, the method is invariant to transformations of the diagnostic scores [1,8]. Because the ROC graphs are based on the true positive rate and the false positive rate, they do not depend on the distribution of both classes [9]. Because of these attractive features, the method has been applied in many different domains. A commonly used method to determine the relative strength of a test is the Area under the Curve (AUC), which is applied to the ROC curve [10].

Despite its popularity, the ROC approach also has a few drawbacks. A ROC curve compares the accumulation of the rate of false negatives with the accumulation of the rate of true positives, given all possible thresholds. Briggs & Zaretzki [1] have stated that ROC curves lack or obscure several quantities that are necessary for the evaluation of the operational effectiveness of diagnostic tests.

As the ROC curve uses all values of the diagnostic instrument, this includes the area of overlap in which the test results can be the most inconclusive. Feinstein [11] criticized the dichotomization approach as inadequate: many clinical decisions are trichotomous rather than dichotomous and many diagnoses are cited as present, uncertain, absent or yes, maybe, no. Although a few proposals for the demarcation of three zones have been published [12–17], dichotomization is still prevalent. Shinkins and Perera [18] conclude that the single decision threshold methods fail to allow for the explicit recognition of diagnostic uncertainty and argue again for explicit identification of patients with an uncertain or inconclusive diagnosis.

### TG-ROC Method

**Fig 1:**
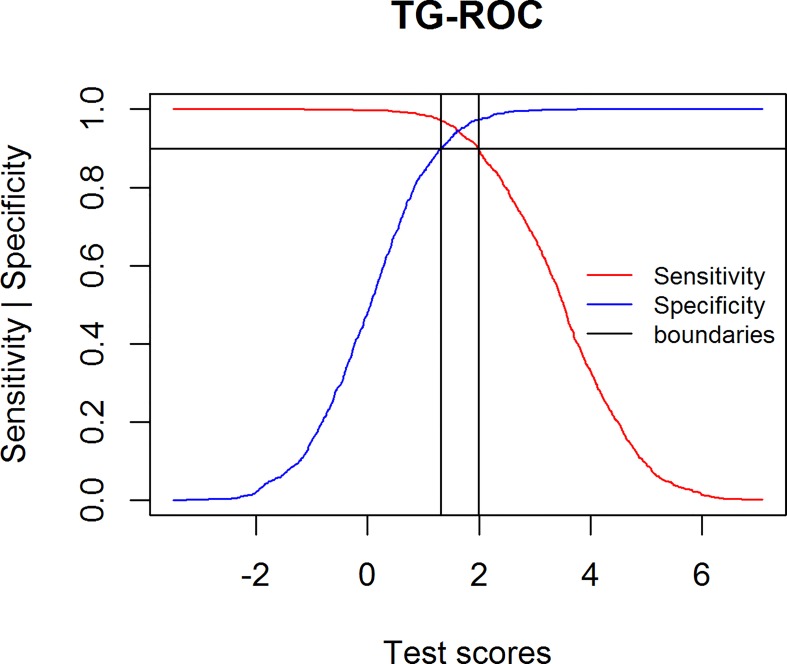
TG-ROC graphic.

The Two-Graph Receiver Operating Characteristic (TG-ROC; [15–17]) approach comes nearest to this identification and is the only one of the trichotomization methods that has acquired a certain popularity.TG-ROC defines a Valid Range of results and considers only these results as valid; the rest of the test scores are considered as being in the Intermediate Range. The TG-ROC method is mainly used for tests that detect the presence of an antigen in a liquid or wet sample, most specifically ELISA (Enzyme-linked Immunosorbent Assay). TG-ROC identifies the two thresholds that have sensitivity and specificity above a pre-selected value (95% or 90%). The two decision thresholds (Se and Sp equal or larger than the pre-selected value) define the two limits of the Intermediate Range. Fig 1 illustrates this: for all possible decision thresholds, both sensitivity and specificity are calculated and plotted in a single graph. The interval between the lower decision threshold and the upper decision threshold identifies the Intermediate Range. Greiner et. al [17] interpreted this as a “borderline range for the clinical interpretation of test results” (p. 123); only scores outside the Intermediate Range are considered valid. The authors claim that “The TG-ROC algorithm warrants a Se and Sp of at least 95% (90% can be optionally selected; other accuracy levels can be evaluated graphically) if only results outside Intermediate Range are considered valid” ([17], p. 130). The goal of the method is therefore maximizing the number of correct decisions by only considering the test scores in the valid range.

There is an interpretational problem with TG-ROC concerning strong tests. For most tests, the lower bound is associated with Se: patients with test scores *higher* than the pre-selected value are diagnosed with the targeted condition, and as a result 90% or 95% of the patients who have the condition are diagnosed correctly (at the cost of low specificity). The upper bound is associated with Sp: patients with test scores *lower* than the pre-selected value are diagnosed without the targeted condition, and as a result 90% or 95% of the patients (dependent on the pre-selected value) who do not have the condition are diagnosed correctly (at the cost of low sensitivity). The interpretational issue is that very strong tests can have an intersection of Se and Sp above the preselected value. As a result, the lower boundary is then associated with Sp and the higher boundary with Se. Greiner at al. [17] discuss these (near) ideal tests and suggest as a solution to consider the Intermediate Range as equal to zero and use a single threshold method instead, as in most cases the range involved is extremely small. Alternatively, the boundaries can be accepted as they are, as the lower boundary is associated with a value of Se that is in fact better than the pre-selected value and, similarly, the upper boundary is associated with a value of Sp that is better than the pre-selected value.

### Uncertain Interval Method

This paper presents a different trichotomization method that uses two decision thresholds to define an interval of uncertainty, in which the test scores are inter-mixed and have a near equal probability of indicating ‘health’ or ‘disease’ and offer little or no information about the presence or absence of the disease. When a test result falls into this interval, any decision is an uncertain one. It is therefore called the Uncertain Interval method. It is expected that a relatively large number of erroneous decision are avoided and, consequently, the rate of correct decisions is expected to improve for the test scores outside this interval.

**Fig 2:**
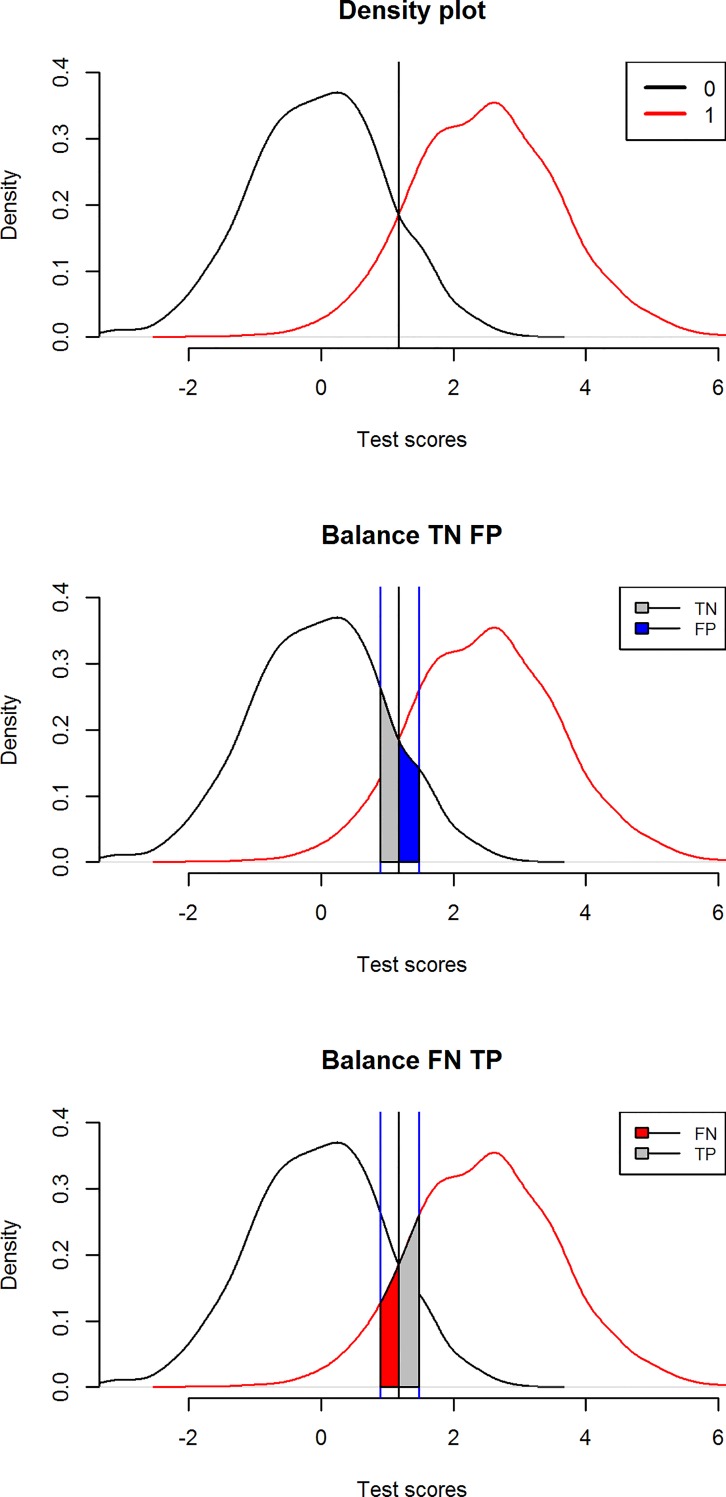
Density of ‘healthy’ (0) and ‘diseased’ population (1) and the definition of an Uncertain Interval with balanced TN and FP and balanced FN and TP.

For a diagnostic test, we distinguish the distributions of patients and non-patients. This is illustrated in Fig 2 for a relatively strong test (AUC = .96). On the left we see the density of the test score distribution of ‘healthy’ people (black line), and ‘diseased’ people (red line). The two distributions intersect at the vertical line. We can also see that the proportion of individuals who really have the targeted condition (‘diseased’) and the proportion of individuals who do not have the targeted condition (‘healthy’) are equal where the two curves meet. At this point of intersection, the test score provides no information on whether an individual is diseased or not.

The basic idea is that we can find an area around the intersection with true negatives (‘healthy’ people with a score below the intersection; grey) to the left of the intersection that is balanced by an area with false positives (‘healthy’ people, with a score above the intersection; blue) to the right. At the same time, we can find an area with true positives (‘diseased’ people with a score above the intersection; grey) to the right of the vertical line that is balanced by an area with false negatives (‘diseased’ people, with a score below the intersection; red) to the left. Clearly, if we find the outer boundaries of these four areas, we have an interval of test scores where FP and TN, as well as FN and TP have almost equal probability. In other words: an interval in which the probability of a correct diagnosis is almost equal to that of a false diagnosis. We therefore expect that the test scores within this uncertain interval cannot provide sufficient evidence to distinguish between individuals with or without the condition.

For the patients outside that interval we expect an improvement of correct decisions, because we expect that a relatively large number of possible false decisions will be found within the Uncertain Interval. If so, the determination of this interval of uncertainty would allow us to prevent unwarranted decisions.

Both the TG-ROC and the Uncertain Interval method define ranges of test-scores that are expected to enable a strong (but not perfect) distinction between the two groups with and without the targeted condition and a third interval in the middle that is expected to offer a weak distinction. The distinction between the two methods is very relevant: TG-ROC defines its Valid Range, with the use of two standard dichotomizations over all test scores and a pre-selected value of either .9 or .95: 1. Sensitivity larger or equal than the pre-selected value and 2. Specificity larger or equal than the pre-selected value. The expectation is that the resulting valid ranges offer the selection of patients with and without the targeted condition with sensitivity, respectively specificity, which is equal or better than the pre-selected value. The Intermediate Range is the range of test scores that remains after the selection of the two Valid Ranges. In contrast, the Uncertain Interval method defines an Uncertain Interval around the intersection of the two distributions, with a low pre-selected value for both Sensitivity and Specificity that is specific for the Uncertain Interval. The pre-selected value has a default of .55. This Uncertain Interval of test scores is expected to be inter-mixed and to offer almost equal test-scores for both groups of patients. Consequently, it is expected that the test scores within this Uncertain Interval do not warrant any distinction between the two groups of patients. The interval outside this Uncertain Interval is called the More Certain Interval (MCI) and is expected to have a superior rate of correct classifications (CCR or Accuracy), because the majority of false classifications are expected to be found in the Uncertain Interval.

The basic question is therefore whether these methods meet their expectations. The first question of this study is, whether the two trichotomization methods do increase the rate of correct decisions when test scores fall in TG-ROC’s Valid Range or outside the identified Uncertain Interval. The second question deals with whether the rates of correct decisions in TG-ROC’s Intermediate Range and within the Uncertain Interval are sufficiently low to withhold or postpone a clinical decision. To answer these questions, we use both a clinical dataset and simulations.

Concerning the practical usefulness of this method, an additional question concerns the possibility of determining the Uncertain Interval and the improvement it allows for tests of various strengths: does the strength of the test influence the possibility of determining an Uncertain Interval? Both strong (AUC > .9) and weak tests (AUC < .7) are suspect. Strong tests may present a strong separation between the two values. This can lead to a small overlap between the two distributions, which may prevent the determination of an Uncertain Interval. Weak tests may be weak over the complete range of test scores. This means that even when an Uncertain Interval can be determined, there is hardly any noticeable improvement in the remaining interval. The strength of tests is manipulated using different distances between the populations with and without the targeted condition, and by varying the difference in standard deviations of the two populations.

In the first part of this study, the Uncertain Interval method is demonstrated on a clinical sample [19] concerning the diagnosis of the severity of prostate cancer. The question this study asks is whether the diagnosis based on pre-surgical diagnostics can be improved. The Uncertain Interval method is compared to various classical methods of decision threshold determination and the results of TG-ROC. The second part is a simulation study that shows the advantages and disadvantages of the proposed method. The method is compared to both the most popular single decision threshold method that maximizes the Youden index and to TG-ROC.

In the following sections, we start with the description of the methods used in the clinical example and the simulation study. The clinical example explains the practical details of the method and provides a first comparison of different methods for the determination of decision thresholds. The simulation study examines the technical qualities of the Uncertain Interval method, compared to a single decision threshold method, the maximized Youden Index, and the alternative trichotomization method TG-ROC. The final part is a discussion of the strengths and weaknesses of the method based on both the results of the simulations and the clinical example. The implementation in R [20] is made available in S1 and S5 Files R-code together with the code to create the figures and Tables (S2 and S3 Files: R-code).

## Methods

### Clinical example

The clinical example is based on the data published by Hosmer and Lemeshow [19], which concerns the diagnosis of the severity of prostate cancer. Their book contains a more complete description of the data and its analysis. The presented results are only intended as an example for the comparison of different methods for decision threshold determination. The targeted condition is the penetration of prostate cancer though the prostate capsule. The predictive model is a combination of tests that can be administered before a surgical intervention. As the individual tests only give a rough indication of the seriousness of the disease, multiple tests have been combined, using logistic regression. In this case, the logistic regression provides a single diagnostic predictor: the risk (probability) of capsular penetration. The standard of this targeted condition is based on surgical intervention, which provides clear results concerning capsular penetration. In this study, 227 patients without this condition are compared to 153 patients with the condition. For comparison, the Uncertain Interval method has been applied to these probabilities, together with a variety of other methods for decision threshold determination.

### Simulation Design

Using simulated data, three methods for decision threshold determination are compared: the maximized Youden index (as the single decision threshold method), the TG-ROC method and the Uncertain Interval method. The comparisons are based on 1000 simulations of the test results of 1000 tested individuals, with 27 models of tests. The objective of the simulations is to describe and compare the differences between the three methods. The main evaluation criterion for comparing the three methods is the rate of correct decisions (CCR). The main criterion for comparing the Uncertain Interval with TG-ROC’s Intermediate Range is the t-test of the mean difference of test scores within the two regions.

In the simulations, tests with a bi-normal distribution are used. Many tests have such a bi-normal distribution, while in other situations test-results can often be sufficiently approximated with the bi-normal model [21].

For creating the 27 test models, three parameters of the ‘healthy’ and the ‘diseased’ population are systematically varied: 1. Mean distance, 2. Standard deviations, and 3 Prevalence. The values of these parameters were based on the work of Somoza [22], who studied the separation of a wide variety of diagnostic tests. These test models are considered as instances of feasible tests. According to general practice, the distribution of ‘healthy’ individuals (D0) is described as a standard normal distribution (M0 = 0, sd0 = 1), while the distribution of the ‘diseased’ population (D1) is allowed to vary in mean (M1 = 3, 2 and 1) and standard deviation (sd1 = .6, 1 and 1.5). Next to these two parameters, the prevalence has been varied (0.5, 0.2 and 0.1) as a lower prevalence may diminish the quality of the estimates. In these simulations, higher scores represent a higher measure of disorder.

These parameter differences result in large differences between the distributions of ‘healthy’ individuals and individuals with the targeted disease. Especially the variance ratio differs greatly, from .36 / 1 to 2.25 / 1. This results in a series of 27 tests that have a considerable variation in the overlap between the distributions: as ΔM increases, the overlap between distributions decreases and the test is more accurate, while a higher sd of D1 flattens its curve, causes more overlap and consequently leads the tests to decrease in discriminatory power. As a result, we have a wide variety of simulated tests which differ in their performance. As the Uncertain Interval method is strictly dependent on the overlap between the two distributions, its performance is expected to be dependent on the overlap.

#### Methods of single decision threshold determination

This paper uses the classic methods as implemented by Lopez-Raton et al. [4]. The simulation study uses the single decision threshold based on the maximum Youden index. The Clinical Example presents a wider range of methods for the determination of single decision thresholds. In addition to the maximized Youden index, the following methods have been applied: maxSe (maximizes sensitivity); maxSp (maximizes specificity); MinPvalue (minimizes p value associated with the statistical χ2-test which measures the association between the marker and the binary result obtained on using the decision threshold); ROC01 (Minimizes distance between the plot of the receiver operating characteristic (ROC) and point (0, 1); SpEqualSe (minimizes the absolute value of Sp–Se); and .5 (the middle of the probability range zero to one). A more complete description of a large range of decision threshold determination methods can be found in [4].

### TG-ROC

Greiner [15] introduced this method as an Excel template. This closed source software enables the selection of two cut-off values that realize a pre-selected level of sensitivity and specificity, respectively. In this way, it realizes an Intermediate Range of test scores that are considered less valid. The TG-ROC method uses both the sensitivities and specificities of all possible decision thresholds. As a criterion for the uncertainty of this interval, the difference between the test-scores has been used. The t-test offers a statistical indicator to show the uncertainty / inconclusiveness of the test results within the Intermediate Range. As the results are expected to be invalid for distinguishing the ‘healthy’ group and the group with the targeted disorder, it may be expected that the mean of the test scores of both groups within the Intermediate Range barely differs and that a t-test would indicate an insignificant difference. An insignificant t-test result indicates that a decision concerning patients within the Intermediate Range of test scores is unwarranted and is better avoided. However, such statistical criterion is also dependent on the number of subjects within the range: in large samples, small differences can lead to significance. It is therefore important to look at the determined difference as well and to evaluate its relevance for practice. The t-test can also be applied to the Uncertain Interval, which enables the comparison of the Uncertainty Interval with TG-ROC’s Intermediate Range.

For TG-ROC’s determination, the R package DiagnosisMed [23] was used. This is easier to use and less limited than the original Excel template. In DiagnosisMed, the parametric method is implemented as a neural network, which may show over-fitting [24]. Comparison of the two software implementations showed that the non-parametric results were similar, while the parametric results showed differences, caused by different implementations. The non-parametric approach has therefore been chosen.

### Uncertain Interval Method

An R function is written for the determination of the upper and lower boundaries of the interval. First, the method determines the point of intersection between the two distributions of individuals with and without the targeted condition. For all possible decision thresholds lower than the intersection, the true negatives (TN) and false negatives (FN) are counted for the interval between the decision threshold and the intersection. Similarly, for decision thresholds higher than the intersection, the true positives (TP) and false positives (FP) are counted between the decision threshold and the intersection. The function then searches for possible combinations of lower and upper limits, while applying a restriction to the ratio of TN and FP and the ratio between FN and TP. Sensitivity and specificity are calculated for each of the candidate intervals. Lastly, candidate Uncertain Intervals are selected if they have specificity and sensitivity below a given value (default .55).

The sensitivity and specificity of scores within the uncertain interval reflect the balance between TP / FN which is equal to Sp / (1 –Sp) and the balance between TN / FP which is equal to Se / (1 –Se). The definition of perfect uncertainty within the Uncertain Interval is unambiguous: correct decisions and false decisions around the intersection are in perfect balance and both Se and Sp are equal to .5. This results in a very small interval around the intersection. In practice, it is desirable to widen this interval. In this study, a default value of .55 has been chosen, which allows for a small amount of positive bias (TP / FN ≤1.22 and TN / FP ≤1.22). If a larger interval is desired, a larger value for the Se and Sp of the Uncertain Interval can be chosen. The value of .55 can be considered a rule of thumb that is not completely arbitrary. In S4 File, Table A is discussed, which concerns the χ^2^-test significance for the possible values of Se or Sp within the Uncertain Interval and the number of individuals with test scores within the Uncertain Interval.

### Mixed Probability Histogram

**Fig 3:**
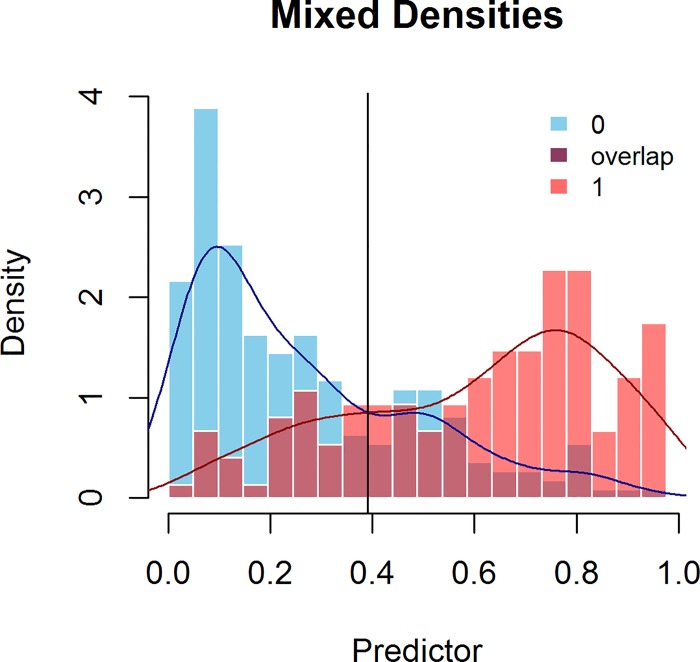
Mixed Probability Histogram for the prediction of capsular penetration in cases of prostate cancer.

Based on a suggestion by Tjur [25], this histogram shows the two overlaid histograms of the probabilities obtained from a logistic regression model for each of the two binary values. It shows the overlap between the two distributions; an example is provided in Fig 3. By default, the histograms are created with the use of 20 bins for all the probability values between zero and one. A test that accurately separates the two binary values shows relatively little overlap between the two distributions. Weak models have much overlap and/or overlap across a wide range of values.

## Results

### Clinical example

As an illustration of the application of this approach as well as its usefulness, we have used the dataset described by Hosmer and Lemeshow [19]. Using logistic regression, the probability of capsular penetration is calculated for the 380 patients, based on a variety of predictors: age, digital rectal examination (DRE), the Prostatic Specific Antigen Value (PSA) and the total Gleason score. The question here is how well capsular penetration can be predicted when pre-surgical diagnostics are used.

The logistic regression model is shown in Table B in S4 File. Various indicators of the predictive value show that the predictive probabilities of the model have intermediate predictive strength: McFadden pseudo R^2^ = .29, Cox-Snell pseudo R^2^ = .32, Nagelkerke pseudo R^2^ = .43. The diagnostic accuracy (AUC = 0.839) of the resulting predictor can be labeled ‘very good’ [10].

Both Table 1 and the histogram of the mixed probabilities (Fig 3) show a predictive problem: the predictions for patients with capsular penetration (1) and the predictions for patients with no capsular penetration (0) cover almost the full range of possible probabilities. Fig 3 shows that there is a relatively large interval in the middle, in which both states are predicted (almost) equally. On the left, the low predictor scores mainly represent patients without capsular penetration, while on the right, the high predictor scores mainly represent patients with capsular penetration

**Table 1:** Summary of the probability distributions of patients with and without capsular penetration.

	Capsular penetration	
	no	yes
**Minimum**	0.000	0.046
**1st Quartile**	0.089	0.418
**Median**	0.196	0.662
**Mean**	0.265	0.607
**3rd Quartile**	0.401	0.805
**Maximum**	0.958	0.976

If we want to know which range of test scores does not allow for distinguishing patients without and with capsular penetration, we select an interval around the point of intersection in which both diagnoses are intermixed and the number of true and false diagnoses of both distributions are (almost) equal. The point of intersection is used as central point of this area.

**Fig 4:**
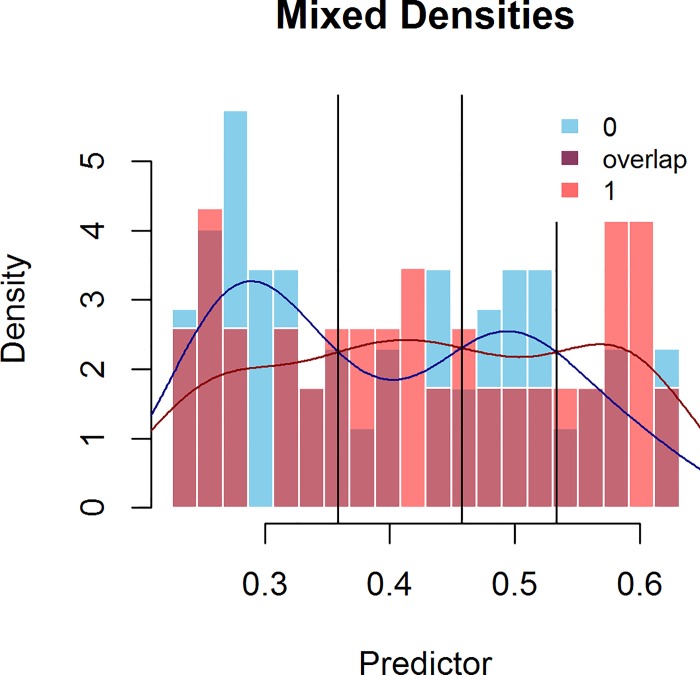
Mixed Probability Histogram for the prediction of capsular penetration for patients within the Uncertain Interval.

The function for calculating this Uncertain Interval returns a rather large range of probabilities between .226 and .632, when using the default (.55) for both the specificity and sensitivity within this interval. Fig 4 shows the histogram of mixed distributions within the range. Clearly, these test scores are intermixed: for each test-score, similar, slightly higher or slightly lower test scores can indicate patients with or without capsular penetration.

Table 2 shows the decision table for all patients, when the Uncertain Interval method is applied. For patients outside the Uncertain Interval, the CCR is .873, Se is .865 and Sp is .879. The majority of false diagnoses is concentrated around the point of intersection and can be found within the Uncertain Interval. Table 3 shows results for patients who have received the diagnosis ‘Uncertain’. In this table, the point of intersection is chosen as the central cut-point and the table shows that in the Uncertain Interval the test scores below and above the intersection are (almost) balanced for patients with and without capsular penetration. These differences are not statistically significant (χ^2^ = .788, df = 1, p = .375). There are 143 patients in the Uncertain Interval, while 87% of the remaining 237 patients outside the Uncertain Interval are classified correctly. The t-test results of the Uncertain Interval show a mean difference between the two distributions of .03 (t = -1.60, df = 115.76, p = .11). Within this interval, it is nigh impossible to distinguish between patients with and without capsular penetration. These 143 patients have test scores that provide little useful information for decision-making. The Uncertain Interval shows both a low sensitivity and specificity close to the maximum desired value (the default of .55).

**Table 2:** Diagnostic decisions based on the Uncertain Interval method.

	Observed Capsular penetration		
Diagnosis	No	Yes	Sum
**No capsular penetration**	124	13	137
**Uncertain**	86	57	143
**Capsular penetration**	17	83	100
**Sum**	227	153	380

**Table 3:** Observed results within the Uncertain Interval.

	Observed Capsular Penetration		
	No	Yes	Sum
**Test score < intersection**	47	26	73
**Test score > intersection**	39	31	70
**Sum**	86	57	143

For the comparison with other methods for the determination of decision thresholds, Table 4 shows the CCR, sensitivity and specificity for various methods of decision threshold determination. The probabilities (between 0 and 1) based on the logistic regression are used as the diagnostic predictor.

**Table 4:** Results of various methods for decision threshold determination applied to the probabilities that are predictive of capsular penetration.

**Trichotomization methods**	**Range**	**n**	**CCR**	**Se**	**Sp**
**More Certain.Interval**	< 0.226 or > 0.632	237	0.873	0.865	0.879
**Uncertain.Interval**	> = 0.226 and < = 0.632	143	0.545	0.544	0.547
**TG-ROC.9 Valid Range**	< 0.236 or > 0.578	260	0.850	0.853	0.848
**TG-ROC.9 Intermediate Range**	> = 0.236 and < = 0.578	120	0.542	0.500	0.566
**TG-ROC.95 Valid Range**	< 0.139 or > 0.703	180	0.894	0.895	0.894
**TG-ROC.95 Intermediate Range**	> = 0.139 and < = 0.703	200	0.625	0.610	0.634
**Dichotomization methods**	**Decision threshold**	**n**	**CCR**	**Se**	**Sp**
**maxSe**	0.046	380	0.458	1.000	0.093
**MinPvalue**	0.107	380	0.582	0.961	0.326
**Youden**	0.343	380	0.753	0.817	0.709
**ROC01**	0.367	380	0.755	0.797	0.727
**SpEqualSe**	0.418	380	0.753	0.752	0.753
**.5**	0.500	380	0.768	0.667	0.837
**maxSp**	0.961	380	0.613	0.039	1.000

n: number of patients diagnosed. For the dichotomization methods this concerns all patients, for the trichotomization methods this concerns the number of patients within the given range.

CCR: Correct Classifications Rate (or Accuracy)

Se: Sensitivity

Sp: Specificity

At the bottom of the table, seven single threshold methods are presented. They show that choosing a lower decision threshold favors sensitivity, while diminishing specificity and vice versa. The method MaxSe offers maximum sensitivity at the cost of a low CCR, the method MaxSp offers maximum specificity, again at the cost of a low CCR. The MinPValue offers high sensitivity, but a low CCR. The other methods (ROC01, Youden, SpEqualSe) offer about equal CCR and both reasonable sensitivity and specificity scores. Splitting the predictions through the middle (decision threshold .5) offers the highest number of correct classifications (CCR = .768) of all single decision threshold methods.

The top of the table shows the results of the Uncertain Interval method. CCR, Se and Sp of the More Certain Interval are higher than those of the single decision threshold methods. False decisions are concentrated in the Uncertain Interval, where the chance of a correct decision is barely more than fifty percent.

TG-ROC with Se and Sp equal to .9, shows an Intermediate Range that is somewhat smaller than the Uncertain Interval and has slightly lower values for CCR and Se. The t-test results for the test scores within TG-ROC’s Intermediate Range are: a mean difference of .016 (t = -.84, df = 97.7, p = .46). It should be observed that for these data neither sensitivity nor specificity reaches the desired value of .9 in the Valid Range.

When TG-ROC is applied with a more stringent restriction (Se and Sp equal to .95), the results are slightly better than those of the Uncertain Interval method: both sensitivity and specificity are higher, but the Intermediate Range is also larger than the Uncertain Interval. TG-ROC’s Intermediate Range has higher values for CCR, Se and Sp. The t-test results reveal a significant difference between the two distributions: a mean difference of 0.105 (t = .450, df = 157.4, p < .001). This shows that there are substantial more correct decisions than false decisions in this Intermediate Range and that the test scores are not as inconclusive as they ideally should be. TG-ROC’s Valid Range shows values for both Se and Sp that are higher than those of the More Certain Interval, but neither sensitivity nor specificity reaches the desired value of .95.

### Simulation Results

Table 5 concerns the comparison of the results of the Maximized Youden threshold, the interval outside the Uncertain Interval (called the More Certain Interval or MCI) and TG-ROC’s Valid Range. Table 5 shows that the simulated tests differ strongly in strength, with AUC varying from .995 (Model 1) to .711 (Model 27). The AUC is lower when the mean distance between the two distributions of individuals with and without the condition is smaller (column M1) and when the sd of the group with the condition is larger (column sd1; the sd of the ‘healthy’ group is 1). The AUC is independent of prevalence (column Pr).

**Table 5:** Comparison of mean results for Youden, More Certain Interval and TG-ROC’s Valid Range over 1000 simulations.

Test model					Youden					More Certain Interval							TG-ROC’s .9 Valid Range					
	M1	sd1	Pr	AUC	Dt	Size	CCR	Sp	Se	Range		Size	CCR	Sp	Se	p.NA	Range		Size	CCR	Sp	Se
1	3	0.6	0.5	0.995	1.762	1000	0.973	0.963	0.982	1.630	1.884	978.298	0.980	0.971	0.989	0.211	1.288	2.234	907.606	0.992	0.986	0.997
2	3	0.6	0.2	0.995	1.792	1000	0.967	0.964	0.982	1.611	1.913	973.544	0.976	0.972	0.991	0.353	1.290	2.235	910.994	0.988	0.986	0.997
3	3	0.6	0.1	0.995	1.818	1000	0.966	0.965	0.982	1.557	1.959	964.639	0.976	0.974	0.993	0.485	1.284	2.227	912.645	0.986	0.985	0.998
4	3	1.0	0.5	0.983	1.479	1000	0.937	0.934	0.939	1.317	1.672	953.579	0.952	0.952	0.953	0.121	1.281	1.721	943.371	0.954	0.954	0.955
5	3	1.0	0.2	0.983	1.509	1000	0.936	0.935	0.939	1.302	1.702	947.291	0.954	0.954	0.956	0.181	1.286	1.712	944.903	0.953	0.953	0.954
6	3	1.0	0.1	0.983	1.529	1000	0.936	0.936	0.941	1.262	1.746	936.832	0.957	0.956	0.961	0.248	1.283	1.703	946.866	0.951	0.951	0.956
7	3	1.5	0.5	0.952	1.379	1000	0.892	0.919	0.865	1.191	1.646	931.592	0.914	0.948	0.880	0.080	1.075	1.285	964.305	0.897	0.897	0.898
8	3	1.5	0.2	0.952	1.400	1000	0.909	0.920	0.867	1.178	1.680	926.921	0.937	0.950	0.884	0.116	1.054	1.294	952.761	0.898	0.897	0.899
9	3	1.5	0.1	0.953	1.419	1000	0.914	0.919	0.870	1.151	1.760	914.425	0.948	0.955	0.888	0.175	1.023	1.312	938.582	0.898	0.897	0.900
10	2	0.6	0.5	0.957	1.101	1000	0.903	0.869	0.936	0.944	1.223	939.696	0.922	0.884	0.959	0.115	1.215	1.297	979.456	0.902	0.901	0.902
11	2	0.6	0.2	0.957	1.115	1000	0.883	0.870	0.938	0.930	1.233	933.181	0.901	0.885	0.963	0.155	1.209	1.297	981.607	0.902	0.902	0.902
12	2	0.6	0.1	0.957	1.126	1000	0.876	0.869	0.940	0.905	1.255	923.116	0.894	0.886	0.968	0.226	1.199	1.306	978.353	0.902	0.902	0.903
13	2	1.0	0.5	0.921	0.990	1000	0.847	0.845	0.849	0.778	1.218	893.797	0.878	0.878	0.878	0.054	0.716	1.278	863.311	0.884	0.884	0.884
14	2	1.0	0.2	0.922	1.010	1000	0.846	0.845	0.851	0.766	1.252	883.811	0.882	0.882	0.883	0.099	0.710	1.279	860.829	0.884	0.884	0.884
15	2	1.0	0.1	0.921	1.023	1000	0.847	0.846	0.855	0.755	1.291	874.058	0.886	0.886	0.888	0.127	0.694	1.283	853.007	0.883	0.883	0.884
16	2	1.5	0.5	0.866	1.071	1000	0.800	0.862	0.738	0.836	1.411	875.578	0.831	0.911	0.751	0.069	0.068	1.281	704.712	0.858	0.840	0.872
17	2	1.5	0.2	0.866	1.098	1000	0.839	0.864	0.740	0.826	1.450	868.674	0.883	0.914	0.756	0.081	0.061	1.283	655.491	0.847	0.838	0.872
18	2	1.5	0.1	0.867	1.100	1000	0.850	0.862	0.747	0.821	1.516	860.434	0.905	0.921	0.757	0.119	0.049	1.28	634.677	0.840	0.834	0.872
19	1	0.6	0.5	0.805	0.348	1000	0.755	0.644	0.866	0.127	0.516	858.506	0.784	0.650	0.917	0.040	0.232	1.279	557.891	0.820	0.855	0.760
20	1	0.6	0.2	0.804	0.369	1000	0.691	0.647	0.865	0.121	0.533	847.606	0.705	0.651	0.919	0.073	0.223	1.281	634.177	0.842	0.855	0.759
21	1	0.6	0.1	0.804	0.378	1000	0.671	0.649	0.868	0.104	0.555	833.390	0.681	0.653	0.925	0.123	0.215	1.284	657.013	0.848	0.854	0.756
22	1	1.0	0.5	0.760	0.487	1000	0.699	0.693	0.705	0.138	0.862	753.587	0.744	0.743	0.745	0.019	-0.280	1.281	489.844	0.795	0.795	0.795
23	1	1.0	0.2	0.760	0.504	1000	0.697	0.694	0.708	0.128	0.915	735.408	0.751	0.752	0.746	0.037	-0.286	1.283	486.346	0.793	0.793	0.794
24	1	1.0	0.1	0.761	0.521	1000	0.698	0.697	0.712	0.131	0.955	730.098	0.760	0.762	0.744	0.061	-0.300	1.282	484.364	0.791	0.790	0.794
25	1	1.5	0.5	0.711	0.878	1000	0.678	0.815	0.542	0.563	1.371	795.511	0.706	0.893	0.516	0.038	-0.920	1.282	402.866	0.751	0.639	0.809
26	1	1.5	0.2	0.711	0.906	1000	0.762	0.816	0.546	0.586	1.444	795.199	0.825	0.902	0.508	0.048	-0.930	1.280	328.479	0.692	0.632	0.810
27	1	1.5	0.1	0.711	0.903	1000	0.786	0.811	0.555	0.580	1.488	791.549	0.866	0.904	0.508	0.076	-0.960	1.281	300.472	0.655	0.617	0.808

M1, sd1: Mean difference and standard deviation ratio between the two distributions

AUC: Area under the Curve

Dt,: decision threshold of the dichotomization method.

Range: lower and upper decision threshold of the trichotomization methods

Size: Mean number of individuals with test scores inside the interval and range respectively

CCR: correct classification rate

Se, sp: Sensitivity and specificity of the test scores inside the interval and range respectively

p.NA: proportion of simulations that did not allow for determination of the Uncertain Interval

#### Youden

The CCR of the Maximized Youden threshold varies between .973 (Model 1 of Table 5) and .671 (Model 21). Its CCR is dependent on all three test parameters: it is higher when the distance between the two distributions is higher (M1), when sd1 is lower and when the prevalence is lower. The influence of prevalence on the CCR is largest when sd1 is smallest. Sp varies from .965 (Model 3) to .644 (Model 19), while Se varies from .982 (Model 1) to .542 (Model 25). Sp and Se are both dependent on the three parameters, but the influence of the standard deviation and the prevalence differs. The position of the Youden decision threshold (Dt) ranges from 1.818 (Model 3) to .348 (Model 19). The general pattern is that results are systematically dependent on the three parameters and increase or decrease when the test is stronger, while the influence of sd1 and Pr differs.

#### More Certain Interval

The CCR of the MCI varies from .980 (Model 1) to .681 (Model 21). As expected, the CCR of the MCI is systematically higher than the CCR of the Maximized Youden threshold. The smallest gain is found for Model 1 (.007), the largest gain is found for Model 27 (.08); the gain increases when the test is weaker. The Sp of the MCI varies from .974 (Model 3) to .65 (Model 19) and is, in all cases, higher than the Sp of the Maximized Youden threshold. The largest gain is found in Model 27 (.09), while the smallest gain is found for Model 20 (.004); these results are found for the tests with the smallest mean difference between the two distributions. The Se of the MCI varies from .993 (Model 3) to .508 (Model 26) and shows no gain compared to the Maximized Youden threshold for models 25, 26 and 27. The largest gain for the remaining twenty-four models is found in Model 21 (.056), while the largest loss in the three models is found in Model 27 (-.047). In most cases, an improvement is found for both Se and Sp. The mean size of the MCI, expressed in the mean number of patients (out of 1000) who have test scores within the MCI, varies from 978.298 (Model 1) to 730.098 (Model 24) and follows the general pattern of being dependent on test strength.

It is expected that the Uncertain Interval method cannot always find an Uncertain Interval. Column p.Na indicates the proportion of simulations in which the Uncertain Interval could not be determined. Roughly, this proportion is largest when the tests are strongest and the distributions therefore have less overlap: the largest proportion of simulations is found for Model 3 (.485), the smallest for Model 22 (.019). The smallest range of test scores in the Uncertain Interval is found for Model 1 (.254), the largest range is found for Model 27 (.907).

#### TG-ROC’s Valid Range

First, a decision was made concerning the interpretational issue of strong tests. For the strongest tests (model 1 to 5), all boundaries of the Valid Range were found to be reversed. For the tests of intermediate strength (models 6 to 12), a part of the samples (ranging from 13% to 99%) showed reversed boundaries. The accompanying tables are Tables C and D in S4 File. When applying the alternative way of dealing with this issue, it became clear that the best results were reached for these strongest tests. Furthermore, the results are then available for all simulated test models. It was therefore decided to apply the alternative interpretation and re-reverse the boundaries and these results are shown in Tables 5 and 6.

**Table 6:** Comparison of mean results for the Uncertain Interval and TG-ROC’s Intermediate Range over 1000 simulations.

Model					Uncertain Interval									TG-ROC’s .9 Intermediate Range							
	M1	sd1	Prev	AUC	Range		Size	CCR	Sp	Se	ΔM	P(t)	P.NA	Range		Size	CCR	Sp	Se	ΔM	P(t)
1	3	0.6	0.5	0.995	1.630	1.884	32.607	0.522	0.522	0.520	0.020	0.013	0.211	1.288	2.234	141.252	0.751	0.801	0.708	0.337	1.000
2	3	0.6	0.2	0.995	1.611	1.913	40.527	0.531	0.536	0.503	0.020	0.005	0.353	1.290	2.235	150.345	0.782	0.804	0.709	0.342	0.999
3	3	0.6	0.1	0.995	1.557	1.959	54.452	0.539	0.542	0.500	0.034	0.002	0.485	1.284	2.227	152.859	0.795	0.808	0.699	0.352	0.953
4	3	1.0	0.5	0.983	1.317	1.672	69.630	0.535	0.535	0.535	0.027	0.041	0.121	1.281	1.721	85.321	0.584	0.599	0.581	0.058	0.373
5	3	1.0	0.2	0.983	1.302	1.702	80.657	0.538	0.541	0.520	0.030	0.018	0.181	1.286	1.712	86.439	0.598	0.610	0.581	0.065	0.305
6	3	1.0	0.1	0.983	1.262	1.746	97.290	0.542	0.545	0.509	0.036	0.008	0.248	1.283	1.703	85.871	0.634	0.651	0.561	0.081	0.203
7	3	1.5	0.5	0.952	1.191	1.646	102.528	0.539	0.539	0.539	0.033	0.060	0.080	1.075	1.285	58.000	0.555	0.632	0.390	0.005	0.062
8	3	1.5	0.2	0.952	1.178	1.680	111.757	0.541	0.544	0.529	0.038	0.038	0.116	1.054	1.294	78.569	0.625	0.659	0.379	0.014	0.121
9	3	1.5	0.1	0.953	1.151	1.760	131.705	0.543	0.546	0.518	0.047	0.018	0.175	1.023	1.312	104.499	0.671	0.692	0.383	0.026	0.098
10	2	0.6	0.5	0.957	0.944	1.223	90.585	0.538	0.538	0.537	0.021	0.059	0.115	1.215	1.297	29.478	0.502	0.494	0.501	0.000	0.047
11	2	0.6	0.2	0.957	0.930	1.233	102.317	0.540	0.543	0.526	0.023	0.024	0.155	1.209	1.297	27.193	0.521	0.542	0.479	0.003	0.101
12	2	0.6	0.1	0.957	0.905	1.255	118.474	0.543	0.546	0.511	0.024	0.004	0.226	1.199	1.306	32.841	0.555	0.586	0.468	0.007	0.100
13	2	1.0	0.5	0.921	0.778	1.218	159.312	0.543	0.543	0.543	0.031	0.144	0.054	0.716	1.278	205.893	0.571	0.572	0.561	0.050	0.408
14	2	1.0	0.2	0.922	0.766	1.252	177.754	0.543	0.545	0.535	0.035	0.072	0.099	0.710	1.279	216.386	0.572	0.570	0.557	0.052	0.306
15	2	1.0	0.1	0.921	0.755	1.291	193.936	0.545	0.547	0.525	0.040	0.034	0.127	0.694	1.283	233.673	0.584	0.582	0.548	0.061	0.228
16	2	1.5	0.5	0.866	0.836	1.411	186.470	0.544	0.543	0.544	0.043	0.214	0.069	0.068	1.281	482.720	0.618	0.697	0.476	0.144	0.903
17	2	1.5	0.2	0.866	0.826	1.450	200.775	0.544	0.546	0.538	0.046	0.096	0.081	0.061	1.283	578.954	0.671	0.698	0.474	0.147	0.714
18	2	1.5	0.1	0.867	0.821	1.516	214.800	0.545	0.547	0.528	0.052	0.047	0.119	0.049	1.28	619.979	0.685	0.697	0.468	0.153	0.441
19	1	0.6	0.5	0.805	0.127	0.516	212.501	0.544	0.544	0.544	0.028	0.234	0.040	0.232	1.279	596.584	0.626	0.464	0.712	0.119	0.968
20	1	0.6	0.2	0.804	0.121	0.533	233.331	0.545	0.547	0.537	0.031	0.137	0.073	0.223	1.281	516.180	0.544	0.465	0.711	0.123	0.941
21	1	0.6	0.1	0.804	0.104	0.555	256.692	0.546	0.548	0.529	0.033	0.056	0.123	0.215	1.284	494.786	0.511	0.469	0.709	0.127	0.803
22	1	1.0	0.5	0.760	0.138	0.862	369.632	0.547	0.547	0.547	0.049	0.408	0.019	-0.280	1.281	766.204	0.591	0.592	0.589	0.181	0.995
23	1	1.0	0.2	0.760	0.128	0.915	404.779	0.547	0.548	0.543	0.054	0.252	0.037	-0.286	1.283	800.307	0.593	0.593	0.590	0.186	0.960
24	1	1.0	0.1	0.761	0.131	0.955	415.585	0.548	0.549	0.538	0.058	0.114	0.061	-0.300	1.282	813.662	0.592	0.592	0.585	0.185	0.805
25	1	1.5	0.5	0.711	0.563	1.371	306.410	0.546	0.546	0.546	0.056	0.353	0.038	-0.920	1.282	957.497	0.591	0.661	0.483	0.195	0.967
26	1	1.5	0.2	0.711	0.586	1.444	313.020	0.546	0.548	0.541	0.062	0.216	0.048	-0.930	1.28	1102.665	0.636	0.661	0.480	0.193	0.808
27	1	1.5	0.1	0.711	0.580	1.488	320.775	0.546	0.548	0.535	0.068	0.089	0.076	-0.960	1.281	1156.022	0.649	0.661	0.478	0.195	0.541

M1, sd1: Mean difference and standard deviation ratio between the two distributions

Range: lower and upper decision threshold

Size: Mean number of individuals with test scores inside the interval and range, respectively

CCR: correct classification rate

Se, sp: Sensitivity and specificity of the test scores inside the interval and range, respectively

P(t): proportion of simulations that showed significant mean differences between the group with and without the targeted condition.

P.NA: proportion of simulations that did not allow for determination of the Uncertain Interval

The CCR of the Valid Range varies from .992 (Model 1) to .655 (Model 27). The CCR of the Valid Range is smaller than for the Maximized Youden threshold for the models 8, 9, 10, 18, 26 and 27. With the exception of model 10, these are all models with low prevalence and large sd of the diseased group. The largest positive difference is found for model 21 (.177), while the largest negative difference is found for model 27 (-.131). The largest Sp for the Valid Range is found for model 1 (.986), the lowest value is found for model 27 (.617). Only for nine models (1, 2, 3, 4, 5, 6, 10, 11, and 12), a higher or equal value than the pre-selected value is found. Compared to the Maximized Youden threshold, a lower value for Sp is found for nine models (7, 8, 9, 16, 17, 18, 25, 26, and 27). The largest positive difference is found for model 19 (.211), the largest negative difference is found for model 27 (-.194). Similar results are found for Se, which ranges from .998 (Model 3) to .756 (Model 21). The same models that show a higher specificity than the preselected value also show a higher value for sensitivity. Six models (10, 11, 12, 19, 20, and 21) show a lower sensitivity than is found for the Maximized Youden threshold. Compared to the Maximized Youden threshold, the maximum gain is found for model 25 (.267), while the maximum loss is found for model 21 (-.112). The size of the Valid Range, expressed in the mean number of patients who have test scores within the valid range has a maximum of 981.6 (Model 11) and a minimum of 300.5 (Model 27). For the Intermediate Range, the smallest range of test scores is found for Model 10 (.082) and the widest range of test scores is found for model 27 (2.241).

#### Comparison MCI and Valid Range

The results in Table 5 are quite different for MCI and TG-ROC’s Valid Range. When comparing CCR, eight of the models show differences smaller than .01 (models 4, 5, 6, 11, 12, 13, 14, and 15). Eight models show a larger CCR improvement for MCI than for the Valid Range (models 7, 8, 9, 10, 17, 18, 26, and 27), and eleven models show a larger improvement for the Valid Range than for MCI (models 1, 2, 3, 16, 19, 20, 21, 22, 23, 24, and 25). The largest positive difference is found for model 27 (.212), the largest negative difference is found for model 21 (-.166). Although in all cases, the ranges of both methods show some overlap, the mean overlap is only .4, with a minimum of .007 (model 10) and a maximum of .823 (model 24). Clearly, the results differ greatly between both methods. To shed more light on the differences, it is preferable to compare the Uncertain Interval with TG-ROC’s Intermediate Range. Table 6 compares both intervals and provides the t-statistic to indicate inconclusiveness.

#### Comparison Uncertain Interval and Intermediate Range

The Uncertain Interval is defined by the Sp and Se of the scores within this Interval. It is expected that both Sp and Se are smaller than the pre-selected value, in this case .55. Table 6 shows that in all cases both Se and Sp are smaller than .55. As a result, the CCR is also systematically smaller than .55. The inconclusiveness is additionally tested with the t-test, and it is expected that the mean differences are small for all tests. Across 1000 simulations, the mean difference between the test scores of the ‘healthy’ and the ‘diseased’ group within the Uncertain Interval varies from .020 (Model 1) to .068 (Model 27). Each of the simulated differences has been tested, and Model 3 gives the smallest proportion of simulations that show significant differences (.002), while Model 22 shows the largest proportion of significant differences (.408). Especially the weaker tests with a large number of individuals in the Uncertain Interval, can show considerable proportions of significant simulations.

In TG-ROC’s Intermediate Range, the rate of correct classifications can be quite high. Only Models 10, 11, 20, and 21 have a CCR smaller than .55. The mean CCR in the Intermediate Range is .615, the Minimal CCR within the Intermediate Range is .502 (Model 10), while the maximum is .795 (Model 3). Similar results are found for Sp and Se: Sp varies from .465 (Model 19) to .808 (Model 3), with only four models (10, 11, 19, 20, and 21) showing an Sp below .55. Se shows a minimum of .379 (Model 8) and a maximum of .717 (Model 19), with a larger range of thirteen models that have an Se below .55: 7, 8, 9, 10, 11, 12, 15, 16, 17, 18, 25, 26, and 27. This result is also reflected when the insignificance of the t-test is applied as an indicator of inconclusiveness: the means between the two test score distributions within the Intermediate Range vary between a minimum of 0 (Model 10) and a maximum of .352 (Model 3), with a mean of .126. The proportion of simulations that show a significant difference varies accordingly: from .047 (Model 10) to 1 (Model 1), with a mean of .561.

In this respect, the position of the Uncertain Interval and the Intermediate Range is relevant. The Uncertain Interval is defined around the intersection of the two distributions where the test scores are inter-mixed, but the Intermediate Range lacks such a definition. In all cases, the Maximized Youden threshold falls within the Uncertain Interval. This is not always the case for the Intermediate Range: for the models 7, 8, 9, 10, 11 and 12 the Maximized Youden threshold falls outside of the Intermediate Range. The overlap between the Uncertain Interval and the Intermediate Range can be very small with a minimum of .007 (Model 10), while in other cases there is some more overlap, up to a maximum of .823 (Model 24).

## Discussion

The main conclusion of this study is that the Uncertain Interval method can offer two advantages: Firstly, it identifies the patients for which the test scores do not allow a diagnosis and a relatively large number of false diagnoses are avoided. Secondly, because these miss-classifications are avoided, the correct classification rates for the remaining group of patients improve.

The results of the clinical data suggest that, in this case, both the Uncertain Interval method and the TG-ROC method offer similar advantages to single decision threshold methods: the identification of the group of patients for whom the test offers insufficient certainty for a diagnosis and improved estimates for the prediction of capsular penetration based on pre-surgical tests. In this example, both trichotomization methods offer similar additional information and an increased CCR in the More Certain Interval and Valid Range, and provide a strengthened foundation for clinical decision-making. Using either of these methods enabled the reduction of the number of false decisions, while maintaining a relatively high level of sensitivity and specificity. It also enables identification of patients for whom the test scores offer little useful information. The difference between the Intermediate Ranges and the Uncertain Interval seems no more than the result of variations in the stringency of restrictions.

The simulations far more clearly show the differences between the two trichotomization methods. The simulation results show that the Uncertain Interval lives up to its expectations: within the Uncertain Interval both Se and Sp are smaller than the pre-selected value (.55) and the number of correct and incorrect classifications are nearly equal. The Uncertain Interval has various attractive properties. 1. For all 27 simulated tests, the differences between the test scores of the two groups are small within the Uncertain Interval. 2. There is a direct relationship between the strength of a test and the size of its Uncertain Interval. 3. The interval outside the Uncertain Interval shows a systematic increase of the CCR for all 27 simulated test scenarios, while improving both Se and Sp for 24 out of 27 cases.

The alternative trichotomization method TG-ROC shows very dissimilar simulation results. Although the method may show desirable results for the strongest tests, it does not do so for the other simulated tests (AUC < .92). TG-ROC’s Valid Range does not always maximize the number of correct decisions by considering only the test scores in the Valid Range. In some cases, both Se and Sp of the Valid Range are found to be smaller than the pre-selected value (in this case .9), which is in contrast to what is claimed. The explanation for these results is straightforward: tests with an AUC < .92 still have a fair amount of inter-mixed test scores in the Valid Range and these inter-mixed test scores reduce Se and Sp. Only the strongest tests with little inter-mixing show the desired improvement of Se and Sp. For the Valid Range only test scores *lower* than the lower bound and *higher* than the upper bound are considered. Although the lower scores will identify healthy patients the best and higher scores will identify patients with the condition the best, and despite the claim of Greiner et al. [17], it is not clear how Se and Sp of the Valid Range relates to the pre-selected values of Se and Sp. The results show that Se and Sp of the Valid Range can be higher or lower than the pre-selected values, and are dependent of the quality of the test. When applying the Maximized Youden Index, Se and Sp over all test scores can be higher.

The simulations show that the Intermediate Ranges and Uncertain Intervals overlap in all cases, but that this overlap can be small. Although the Intermediate Range is expected to define an interval of less valid test scores, it does not show a clear relationship with the area of overlap around the intersection, where the least valid test scores can be expected. The Intermediate Range is not always sufficiently inconclusive compared to the Uncertain Interval, while the Valid Range offers not always the expected Sensitivity and Specificity, that is, higher than the pre-selected value of .9 or .95. The conclusion here is that especially TG-ROC’s Intermediate Range has ill-defined properties and that TG-ROC’s Valid Range does not always meet expectations. The TG-ROC simulation results show that the idea of selecting only the most discriminative scores, while leaving out intermediate scores does not necessarily lead to the desired improvement in decision-making.

The Uncertain Interval method indicates that a weak test does impede the unambiguous diagnosis of a relatively large number of patients: a weak test has a large Uncertain Interval. However, it should be clear that patients with test scores within the Uncertain Interval are in no way discarded. Instead, they receive the diagnosis ‘Uncertain’, simply because the available test scores do not allow for a positive or a negative decision. Patients, whose results fall within the Uncertain Interval, are about as likely as not to have the disease. For these patients further testing, or awaiting further developments, is a better choice than a positive or negative diagnosis. In this study, individuals with test scores within the Uncertain Interval are not considered to be classified correctly. This is debatable: one could argue that the diagnosis ‘Uncertain’ is the most appropriate diagnosis for these patients. Although this result may be inconvenient, it is the best classification that is available for these patients, given the available test results.

Although the applicability of the Uncertain Interval method has been demonstrated for a wide range of tests, the simulations also show that in 47% to 2% of the simulated samples no solution was found. The percentage of samples that offered no solution is especially high in case of a strong test with low overlap. In the case of a strong test, it may be impossible to define an Uncertain Interval. For the weakest tests (AUC = .71) within the range of realistic, simulated tests, the Uncertain Interval method worked appropriately, and clearly improved accuracy. The Uncertain Interval method should be applied with careful examination of the results. The functions provided for the diagnosis of both the More Certain Interval and the Uncertain Interval offer sufficient possibilities for acceptance or rejection of the resulting interval.

Several important questions are not answered in this study. An important question is how large a sample has to be for application of the Uncertain Interval method. In general, it is difficult to answer this question with any degree of accuracy. It is recommended for most decision threshold methods that the sample is ‘large enough’ with ‘larger than 200’ as a possible rule of thumb. For a statistical test that concerns sample differences, a sufficient sample size would be determined by the power to find a statistically significant difference between the two subsamples, which is a function of a population parameter: the true difference between the two subpopulations. The determination of a sufficient sample size for determining a decision threshold that reduces false individual diagnoses is quite a different problem. The main difference is that a decision threshold is a function of the true status of the individuals and not a function of a population parameter. The specification of a sufficient sample size is therefore more complicated. Yet another question is how well this method works when test data have other distributions than examined here. The current simulation results do not allow for generalization beyond bi-normal distributed test scores, although the clinical example demonstrates that the method can be applied to the probabilities obtained by logistic regression. Another important question concerns the stability of the results of the Uncertain Interval method. Of course, like all statistics, its results are dependent on sample differences. However, in this case, the most relevant question is how stable the method results are for repeated measures within individuals. This last question requires different datasets than those used in this study. There are also some fundamental questions to be asked. The basic idea of this method is to define an interval of test scores that is uncertain or inconclusive, while ‘uncertain’ is further specified as inter-mixed scores that have small differences. The inter-mixed scores are specified as being positioned around the intersection of both distributions and this can be inspected visually. However, a better statistical definition of ‘inter-mixedness’ would be preferable. From a clinical point of view, another question to be resolved is whether the Uncertain Intervals sufficiently indicate the patients with results that are considered as inconclusive in the field. In different clinical situations, smaller or larger intervals may be desired and a weaker or stronger restriction may be desired than the default .55 for both Sensitivity and Specificity. The current results show the applicability of the method, but there is room for improvements. This requires further research, which is hopefully sufficiently stimulated by giving complete transparency about the method and its implementation.

## Supporting Information

S1 File The function ‘uncertain.interval’ is used for the determination of the Uncertain Interval. The function ‘quality.threshold’ can be applied both to classical single decision thresholds and to the two decision thresholds of the More Certain Interval. The function ‘quality.threshold.uncertain’ does the same for the Uncertain Interval and applies the χ^2^- test to the difference between TN and FP and to the difference between FN and TP. The function ‘Pseudo.R2’ calculates various pseudo *R*^*2*^ indices for logistic regression models. The function ‘plotMPH’ draws the Mixed Probability Histogram.
(R) Click here for additional data file.

S2 File This file offers the R code for the production of the tables and figures in this paper. (R)

S3 File This file produces Tables 5 and 6. (R)

S4 File Supplementary tables A, B, C and D.
(DOCX)

S5 File Selected R-functions from the DiagnosisMed package. (R)
