# Supplementary material for: Interval of Uncertainty: An Alternative Approach for the Determination of Decision Thresholds, with an Illustrative Application for the Prediction of Prostate Cancer
Source: PLoS One. 2016 Nov 9;11(11):e0166007. doi: 10.1371/journal.pone.0166007 (PMC5102386; doi:10.1371/journal.pone.0166007)
Supplement: S4 File — Supplementary tables A, B, C and D. (DOCX) [file pone.0166007.s004.docx]

# S4 Supplementary Tables

## Discussion of table A: the choice of Se or Sp values

The fact that the Uncertain Interval is positioned around the intersection of the two distributions and falls within the interval where the two distributions overlap, guarantees that the scores of the healthy and the diseased sample are inter-mixed. To define the size of this interval, the ratios of TN / FP and TP / FN are balanced. The first ratio equals Sp / (1 - Sp) and the second ratio equals Se / (1 – Se). For the uncertain interval around the intersection, a value of Sp or Se between .50 and .60 seems reasonable to balance TN / FP and TP / FN. That is to say, these values represent balance or equality (.5) or, for values larger than .5 but smaller than .6, near equality allowing for some positive bias. The value of .55 seems to represent a reasonable choice. However, at this moment, little more than a rule of thumb can be provided.

Table A shows the resulting p value of the χ^2^-test when comparing the equality either of the TN and FP, or TP and FN, when considering the size of the Uncertain Interval (expressed as number of patients with test scores in the interval) and the values of Se or Sp of the test scores in the Uncertain Interval. Given a reasonably sized sample, some positive bias may still represent a non-significant difference between the means of the test scores. When Se and Sp are both .55, the difference between TN and FP and between TP and FN is not significant, not even when the size of the sample within the Uncertain Interval is as large as 350 (p = 0.054). The R code to generate this table is provided in supplemental file S1.

When Se or Sp is set at .5, and an Uncertain Interval is found, the theoretical sample size within the Uncertain Interval can be indefinitely large without leading to statistical significance, but in practice the associated Uncertain Interval will be small. When a value larger than .5 is chosen, the Uncertain Interval becomes larger.

**Table A: χ^2^-test significance given Sensitivity or Specificity and count of test scores within the Uncertain Interval.**

|  | **Sensitivity or Specificity** | | | | | | | | | | |
| --- | --- | --- | --- | --- | --- | --- | --- | --- | --- | --- | --- |
| **Size** | **0.5** | **0.51** | **0.52** | **0.53** | **0.54** | **0.55** | **0.56** | **0.57** | **0.58** | **0.59** | **0.6** |
| 10 | 1 | 1 | 1 | 1 | 1 | 0.527 | 0.527 | 0.527 | 0.527 | 0.527 | 0.527 |
| 30 | 1 | 1 | 0.715 | 0.715 | 0.715 | 0.715 | 0.465 | 0.465 | 0.465 | 0.273 | 0.273 |
| 50 | 1 | 0.777 | 0.777 | 0.777 | 0.572 | 0.396 | 0.396 | 0.258 | 0.258 | 0.157 | 0.157 |
| 70 | 1 | 0.811 | 0.811 | 0.633 | 0.473 | 0.473 | 0.339 | 0.232 | 0.151 | 0.151 | 0.094 |
| 90 | 1 | 0.833 | 0.673 | 0.527 | 0.399 | 0.292 | 0.292 | 0.206 | 0.14 | 0.092 | 0.058 |
| 110 | 1 | 0.849 | 0.703 | 0.567 | 0.446 | 0.253 | 0.182 | 0.127 | 0.086 | 0.057 | 0.036 |
| 130 | 1 | 0.861 | 0.599 | 0.483 | 0.38 | 0.219 | 0.161 | 0.114 | 0.079 | 0.035 | 0.023 |
| 150 | 1 | 0.870 | 0.624 | 0.414 | 0.327 | 0.253 | 0.142 | 0.072 | 0.05 | 0.034 | 0.014 |
| 170 | 1 | 0.759 | 0.645 | 0.443 | 0.283 | 0.167 | 0.125 | 0.066 | 0.032 | 0.021 | 0.009 |
| 190 | 1 | 0.772 | 0.562 | 0.384 | 0.246 | 0.147 | 0.11 | 0.059 | 0.03 | 0.014 | 0.006 |
| 210 | 1 | 0.783 | 0.581 | 0.408 | 0.27 | 0.129 | 0.073 | 0.038 | 0.019 | 0.009 | 0.004 |
| 230 | 1 | 0.792 | 0.51 | 0.356 | 0.235 | 0.114 | 0.065 | 0.035 | 0.018 | 0.006 | 0.002 |
| 250 | 1 | 0.704 | 0.527 | 0.376 | 0.206 | 0.100 | 0.058 | 0.023 | 0.011 | 0.004 | 0.002 |
| 270 | 1 | 0.715 | 0.543 | 0.33 | 0.181 | 0.114 | 0.051 | 0.021 | 0.007 | 0.003 | 0.001 |
| 290 | 1 | 0.725 | 0.481 | 0.291 | 0.159 | 0.078 | 0.046 | 0.019 | 0.007 | 0.002 | 0.001 |
| 310 | 1 | 0.733 | 0.496 | 0.307 | 0.173 | 0.088 | 0.031 | 0.012 | 0.005 | 0.001 | 0 |
| 330 | 1 | 0.741 | 0.441 | 0.271 | 0.152 | 0.061 | 0.028 | 0.011 | 0.004 | 0.001 | 0 |
| 350 | 1 | 0.748 | 0.454 | 0.24 | 0.134 | 0.054 | 0.025 | 0.008 | 0.003 | 0.001 | 0 |
| 370 | 1 | 0.677 | 0.467 | 0.253 | 0.119 | 0.048 | 0.022 | 0.007 | 0.002 | 0.001 | 0 |
| 390 | 1 | 0.685 | 0.418 | 0.224 | 0.105 | 0.043 | 0.02 | 0.006 | 0.002 | 0 | 0 |
| 410 | 1 | 0.693 | 0.429 | 0.236 | 0.114 | 0.038 | 0.014 | 0.004 | 0.001 | 0 | 0 |

The range of significant values is marked in red; one might choose to avoid these. However, statistical non-significance should not be decisive for acceptance of a value. In the end, the acceptable uncertainty (defined here as an acceptable balance between TN / FP and TP / FN, needs to be defined in practice. However, establishing the amount of positive bias to allow is far from easy and a statistical definition may therefore be a start. Depending on the actual diagnosis, a larger or smaller Uncertain Interval may be desired. Furthermore, when the two distributions of patients with and without the targeted condition show large differences in variance, slight differences between the values of Se and Sp can be considered.

2. **Table B: Logistic regression model for prediction of prostatic capsular penetration**

|  | **B** | **SE B** | **z value** | **Pr(>\|z\|)** |
| --- | --- | --- | --- | --- |
| **(Intercept)** | -10.59 | 3.24 | -3.27 | 0.00 |
| **age** | 0.02 | 0.04 | 0.50 | 0.61 |
| **DRE** |  |  |  |  |
| **DRE2** | 7.01 | 3.69 | 1.90 | 0.06 |
| **DRE3** | -0.71 | 3.90 | -0.18 | 0.86 |
| **DRE4** | 1.85 | 5.23 | 0.35 | 0.72 |
| **PSA** |  |  |  |  |
| **2.5-4.4** | 0.40 | 0.69 | 0.58 | 0.56 |
| **4.5-6.4** | 0.50 | 0.67 | 0.76 | 0.45 |
| **6.5-8.4** | 1.52 | 0.64 | 2.37 | 0.02 |
| **8.5-10.4** | 0.73 | 0.69 | 1.06 | 0.29 |
| **10.5-12.4** | -0.14 | 0.79 | -0.18 | 0.86 |
| **12.5-20.4** | 1.05 | 0.66 | 1.59 | 0.11 |
| **20.5-140** | 1.55 | 0.66 | 2.36 | 0.02 |
| **Gleason** | 1.06 | 0.17 | 6.06 | 0.00 |
| **Age: DRE** |  |  |  |  |
| **age:DRE2** | -0.09 | 0.05 | -1.73 | 0.08 |
| **age:DRE3** | 0.03 | 0.06 | 0.60 | 0.55 |
| **age:DRE4** | 0.00 | 0.08 | -0.05 | 0.96 |

Table B Notes:

This table is based on data published in Hosmer and Lemeshow [19]. Suggestions for its analysis, discussed in H & L., have been used, but the analysis has been created with the use of R.

For this analysis, the results of the digital rectal exam (DRE) are divided into the following categories: 1 No Nodule, 2 Unilobar Nodule Left, 3 Unilobar Nodule Right, 4 Bilobar Nodule, with the first category as a reference.

For the PSA (prostate specific antigen level) the following levels are used: [0, 2.4], [2.5-4.4], [4.5-6.4], [6.5-8.4], [8.5-10.4], [10.5-12.4], [12.5-20.4], [20.5-140], with the first category as a reference. The interaction between age and the results of the digital rectal exam is represented by age: DRE, with the interaction of the first category of DRE with age as a reference.

The first column gives the unstandardized B estimates, the column labeled z value is part of the Wald test that tests whether the estimate of the model coefficient is equal to 0, and the last column shows the corresponding significance of each estimate.

The model has a Null deviance of 512.29 (df = 379), a Residual deviance of 366.08 (df = 364), which shows that the model offers an improvement of 146.21 at the cost of 15 df. The AIC is 398.08.

**Simulation results when TG-ROC boundaries for strong tests are unavailable**

**Table C: Comparison of mean results for Youden, More Certain Interval and TG-ROC’s Valid Range over 1000 simulations**

| **Test models** | | | | | **Youden** | | | | | **More Certain Area** | | | | | | | **TG-ROC’s .9 Valid Range** | | | | | | |
| --- | --- | --- | --- | --- | --- | --- | --- | --- | --- | --- | --- | --- | --- | --- | --- | --- | --- | --- | --- | --- | --- | --- | --- |
|  | **M1** | **sd1** | **Prev** | **AUC** | **DT** | **Size** | **CCR** | **Sp** | **Se** | **Range** | | **Size** | **CCR** | **Sp** | **Se** | **P.NA** | **Range** | | **Size** | **CCR** | **Sp** | **Se** | **P.NA** |
| **1** | 3 | 0.6 | 0.5 | 0.995 | 1.762 | 1000 | 0.973 | 0.963 | 0.982 | 1.63 | 1.884 | 978.298 | 0.98 | 0.971 | 0.989 | 0.211 | - | - | - | - | - | - | 1.000 |
| **2** | 3 | 0.6 | 0.2 | 0.995 | 1.792 | 1000 | 0.967 | 0.964 | 0.982 | 1.611 | 1.913 | 973.544 | 0.976 | 0.972 | 0.991 | 0.353 | - | - | - | - | - | - | 1.000 |
| **3** | 3 | 0.6 | 0.1 | 0.995 | 1.819 | 1000 | 0.967 | 0.965 | 0.981 | 1.557 | 1.959 | 964.639 | 0.976 | 0.974 | 0.993 | 0.485 | - | - | - | - | - | - | 1.000 |
| **4** | 3 | 1 | 0.5 | 0.983 | 1.479 | 1000 | 0.937 | 0.934 | 0.939 | 1.317 | 1.672 | 953.579 | 0.952 | 0.952 | 0.953 | 0.121 | - | - | - | - | - | - | 1.000 |
| **5** | 3 | 1 | 0.2 | 0.983 | 1.51 | 1000 | 0.936 | 0.935 | 0.939 | 1.302 | 1.702 | 947.291 | 0.954 | 0.954 | 0.956 | 0.181 | 1.266 | 1.322 | 996.000 | 0.900 | 0.901 | 0.894 | 0.999 |
| **6** | 3 | 1 | 0.1 | 0.983 | 1.53 | 1000 | 0.936 | 0.936 | 0.941 | 1.262 | 1.746 | 936.832 | 0.957 | 0.956 | 0.961 | 0.248 | 1.289 | 1.339 | 990.286 | 0.900 | 0.900 | 0.899 | 0.986 |
| **7** | 3 | 1.5 | 0.5 | 0.952 | 1.379 | 1000 | 0.892 | 0.919 | 0.865 | 1.191 | 1.646 | 931.592 | 0.914 | 0.948 | 0.88 | 0.08 | 1.066 | 1.287 | 962.384 | 0.896 | 0.896 | 0.897 | 0.068 |
| **8** | 3 | 1.5 | 0.2 | 0.952 | 1.4 | 1000 | 0.909 | 0.92 | 0.867 | 1.178 | 1.68 | 926.921 | 0.937 | 0.95 | 0.884 | 0.116 | 1.025 | 1.285 | 948.229 | 0.895 | 0.894 | 0.896 | 0.130 |
| **9** | 3 | 1.5 | 0.1 | 0.953 | 1.419 | 1000 | 0.914 | 0.919 | 0.87 | 1.151 | 1.76 | 914.425 | 0.948 | 0.955 | 0.888 | 0.175 | 0.965 | 1.289 | 929.203 | 0.893 | 0.892 | 0.896 | 0.189 |
| **10** | 2 | 0.6 | 0.5 | 0.957 | 1.101 | 1000 | 0.903 | 0.869 | 0.936 | 0.944 | 1.223 | 939.696 | 0.922 | 0.884 | 0.959 | 0.115 | 1.220 | 1.314 | 975.772 | 0.897 | 0.898 | 0.896 | 0.310 |
| **11** | 2 | 0.6 | 0.2 | 0.957 | 1.116 | 1000 | 0.883 | 0.87 | 0.938 | 0.93 | 1.233 | 933.181 | 0.901 | 0.885 | 0.963 | 0.155 | 1.198 | 1.299 | 978.614 | 0.898 | 0.898 | 0.896 | 0.298 |
| **12** | 2 | 0.6 | 0.1 | 0.957 | 1.126 | 1000 | 0.876 | 0.869 | 0.94 | 0.905 | 1.255 | 923.116 | 0.894 | 0.886 | 0.968 | 0.226 | 1.176 | 1.299 | 974.676 | 0.898 | 0.898 | 0.896 | 0.286 |
| **13** | 2 | 1 | 0.5 | 0.921 | 0.99 | 1000 | 0.847 | 0.845 | 0.849 | 0.778 | 1.218 | 893.797 | 0.878 | 0.878 | 0.878 | 0.054 | 0.716 | 1.278 | 863.311 | 0.884 | 0.884 | 0.884 | 0 |
| **14** | 2 | 1 | 0.2 | 0.922 | 1.01 | 1000 | 0.846 | 0.845 | 0.851 | 0.766 | 1.252 | 883.811 | 0.882 | 0.882 | 0.883 | 0.099 | 0.710 | 1.279 | 860.829 | 0.884 | 0.884 | 0.884 | 0 |
| **15** | 2 | 1 | 0.1 | 0.921 | 1.024 | 1000 | 0.847 | 0.846 | 0.855 | 0.755 | 1.291 | 874.058 | 0.886 | 0.886 | 0.888 | 0.127 | 0.694 | 1.283 | 853.007 | 0.883 | 0.883 | 0.884 | 0 |
| **16** | 2 | 1.5 | 0.5 | 0.866 | 1.071 | 1000 | 0.8 | 0.862 | 0.738 | 0.836 | 1.411 | 875.578 | 0.831 | 0.911 | 0.751 | 0.069 | 0.068 | 1.281 | 704.712 | 0.858 | 0.840 | 0.872 | 0 |
| **17** | 2 | 1.5 | 0.2 | 0.866 | 1.097 | 1000 | 0.839 | 0.863 | 0.741 | 0.826 | 1.45 | 868.674 | 0.883 | 0.914 | 0.756 | 0.081 | 0.061 | 1.283 | 655.491 | 0.847 | 0.838 | 0.872 | 0 |
| **18** | 2 | 1.5 | 0.1 | 0.867 | 1.1 | 1000 | 0.85 | 0.862 | 0.747 | 0.821 | 1.516 | 860.434 | 0.905 | 0.921 | 0.757 | 0.119 | 0.049 | 1.280 | 634.677 | 0.840 | 0.834 | 0.872 | 0 |
| **19** | 1 | 0.6 | 0.5 | 0.805 | 0.348 | 1000 | 0.755 | 0.644 | 0.866 | 0.127 | 0.516 | 858.506 | 0.784 | 0.65 | 0.917 | 0.04 | 0.232 | 1.279 | 557.891 | 0.820 | 0.855 | 0.760 | 0 |
| **20** | 1 | 0.6 | 0.2 | 0.804 | 0.369 | 1000 | 0.691 | 0.647 | 0.865 | 0.121 | 0.533 | 847.606 | 0.705 | 0.651 | 0.919 | 0.073 | 0.223 | 1.281 | 634.177 | 0.842 | 0.855 | 0.759 | 0 |
| **21** | 1 | 0.6 | 0.1 | 0.804 | 0.378 | 1000 | 0.671 | 0.649 | 0.868 | 0.104 | 0.555 | 833.39 | 0.681 | 0.653 | 0.925 | 0.123 | 0.215 | 1.284 | 657.013 | 0.848 | 0.854 | 0.756 | 0 |
| **22** | 1 | 1 | 0.5 | 0.76 | 0.487 | 1000 | 0.699 | 0.693 | 0.705 | 0.138 | 0.862 | 753.587 | 0.744 | 0.743 | 0.745 | 0.019 | -0.280 | 1.281 | 489.844 | 0.795 | 0.795 | 0.795 | 0 |
| **23** | 1 | 1 | 0.2 | 0.76 | 0.504 | 1000 | 0.697 | 0.694 | 0.708 | 0.128 | 0.915 | 735.408 | 0.751 | 0.752 | 0.746 | 0.037 | -0.286 | 1.283 | 486.346 | 0.793 | 0.793 | 0.794 | 0 |
| **24** | 1 | 1 | 0.1 | 0.761 | 0.522 | 1000 | 0.699 | 0.697 | 0.712 | 0.131 | 0.955 | 730.098 | 0.76 | 0.762 | 0.744 | 0.061 | -0.300 | 1.282 | 484.364 | 0.791 | 0.790 | 0.794 | 0 |
| **25** | 1 | 1.5 | 0.5 | 0.711 | 0.878 | 1000 | 0.678 | 0.815 | 0.542 | 0.563 | 1.371 | 795.511 | 0.706 | 0.893 | 0.516 | 0.038 | -0.920 | 1.282 | 402.866 | 0.751 | 0.639 | 0.809 | 0 |
| **26** | 1 | 1.5 | 0.2 | 0.711 | 0.905 | 1000 | 0.762 | 0.816 | 0.546 | 0.586 | 1.444 | 795.199 | 0.825 | 0.902 | 0.508 | 0.048 | -0.930 | 1.280 | 328.479 | 0.692 | 0.632 | 0.810 | 0 |
| **27** | 1 | 1.5 | 0.1 | 0.711 | 0.902 | 1000 | 0.785 | 0.811 | 0.556 | 0.58 | 1.488 | 791.549 | 0.866 | 0.904 | 0.508 | 0.076 | -0.960 | 1.281 | 300.472 | 0.655 | 0.617 | 0.808 | 0 |

Note: TG-ROC boundaries for strong tests are unavailable

Table C legend:

M1, sd1: Mean difference and standard deviation ratio between the two distributions

AUC: Area under the Curve

Dt,: decision threshold of the Youden dichotomization method.

Range: lower and upper decision threshold of the trichotomization methods

Size: Mean number of individuals with test scores inside the interval and range respectively

CCR: correct classification rate

Se, sp: Sensitivity and specificity of the test scores inside the interval and range respectively

P.NA: proportion of simulations that did not allow for determination of the Uncertain Interval or Intermediate Range

Note: These results are equal to those of table 5, except the non-availability of TG-ROC’s Ranges for the stronger tests (column P.NA).

**Table D: Comparison of mean results for the Uncertain Interval and TG-ROC’s Intermediate Range over 1000 simulations**

| **Test Models** | | | | | **Uncertain Interval** | | | | | | | | | **TG-ROC’s .9 Intermediate Range** | | | | | | | | |
| --- | --- | --- | --- | --- | --- | --- | --- | --- | --- | --- | --- | --- | --- | --- | --- | --- | --- | --- | --- | --- | --- | --- |
|  | **M1** | **sd1** | **Prev** | **AUC** | **Ranges** | | **Size** | **CCR** | **Sp** | **Se** | **ΔM** | **P(t)** | **P.NA** | **Range** | | **Size** | **CCR** | **Sp** | **Se** | ΔM | **P(t)** | **P.NA** |
| 1 | 3 | 0.6 | 0.5 | 0.995 | 1.63 | 1.884 | 32.607 | 0.522 | 0.522 | 0.52 | 0.02 | 0.013 | 0.211 | - | - | - | - | - | - | - | - | 1 |
| 2 | 3 | 0.6 | 0.2 | 0.995 | 1.611 | 1.913 | 40.527 | 0.531 | 0.536 | 0.503 | 0.02 | 0.005 | 0.353 | - | - | - | - | - | - | - | - | 1 |
| 3 | 3 | 0.6 | 0.1 | 0.995 | 1.557 | 1.959 | 54.452 | 0.539 | 0.542 | 0.5 | 0.034 | 0.002 | 0.485 | - | - | - | - | - | - | - | - | 1 |
| 4 | 3 | 1 | 0.5 | 0.983 | 1.317 | 1.672 | 69.63 | 0.535 | 0.535 | 0.535 | 0.027 | 0.041 | 0.121 | - | - | - | - | - | - | - | - | 1 |
| 5 | 3 | 1 | 0.2 | 0.983 | 1.302 | 1.702 | 80.657 | 0.538 | 0.541 | 0.52 | 0.03 | 0.018 | 0.181 | 1.266 | 1.322 | 5 | 0 | 0 | 0 | -0.04 | - | 0.999 |
| 6 | 3 | 1 | 0.1 | 0.983 | 1.262 | 1.746 | 97.29 | 0.542 | 0.545 | 0.509 | 0.036 | 0.008 | 0.248 | 1.289 | 1.339 | 16.929 | 0.84 | 0.846 | 0.208 | -0.01 | 0 | 0.986 |
| 7 | 3 | 1.5 | 0.5 | 0.952 | 1.191 | 1.646 | 102.528 | 0.539 | 0.539 | 0.539 | 0.033 | 0.06 | 0.08 | 1.066 | 1.287 | 61.232 | 0.555 | 0.634 | 0.375 | 0.004 | 0.065 | 0.068 |
| 8 | 3 | 1.5 | 0.2 | 0.952 | 1.178 | 1.68 | 111.757 | 0.541 | 0.544 | 0.529 | 0.038 | 0.038 | 0.116 | 1.025 | 1.285 | 86.537 | 0.64 | 0.664 | 0.355 | 0.013 | 0.115 | 0.13 |
| 9 | 3 | 1.5 | 0.1 | 0.953 | 1.151 | 1.76 | 131.705 | 0.543 | 0.546 | 0.518 | 0.047 | 0.018 | 0.175 | 0.965 | 1.289 | 120.94 | 0.676 | 0.686 | 0.337 | 0.024 | 0.097 | 0.189 |
| 10 | 2 | 0.6 | 0.5 | 0.957 | 0.944 | 1.223 | 90.585 | 0.538 | 0.538 | 0.537 | 0.021 | 0.059 | 0.115 | 1.22 | 1.314 | 33.583 | 0.531 | 0.398 | 0.568 | -0.001 | 0.04 | 0.31 |
| 11 | 2 | 0.6 | 0.2 | 0.957 | 0.93 | 1.233 | 102.317 | 0.54 | 0.543 | 0.526 | 0.023 | 0.024 | 0.155 | 1.198 | 1.299 | 30.547 | 0.506 | 0.445 | 0.547 | 0.001 | 0.104 | 0.298 |
| 12 | 2 | 0.6 | 0.1 | 0.957 | 0.905 | 1.255 | 118.474 | 0.543 | 0.546 | 0.511 | 0.024 | 0.004 | 0.226 | 1.176 | 1.299 | 37.154 | 0.514 | 0.476 | 0.541 | 0.003 | 0.082 | 0.286 |
| 13 | 2 | 1 | 0.5 | 0.921 | 0.778 | 1.218 | 159.312 | 0.543 | 0.543 | 0.543 | 0.031 | 0.144 | 0.054 | 0.716 | 1.278 | 205.893 | 0.571 | 0.572 | 0.561 | 0.05 | 0.408 | 0 |
| 14 | 2 | 1 | 0.2 | 0.922 | 0.766 | 1.252 | 177.754 | 0.543 | 0.545 | 0.535 | 0.035 | 0.072 | 0.099 | 0.71 | 1.279 | 216.387 | 0.572 | 0.57 | 0.557 | 0.052 | 0.306 | 0 |
| 15 | 2 | 1 | 0.1 | 0.921 | 0.755 | 1.291 | 193.936 | 0.545 | 0.547 | 0.525 | 0.04 | 0.034 | 0.127 | 0.694 | 1.283 | 233.673 | 0.584 | 0.582 | 0.548 | 0.061 | 0.228 | 0 |
| 16 | 2 | 1.5 | 0.5 | 0.866 | 0.836 | 1.411 | 186.47 | 0.544 | 0.543 | 0.544 | 0.043 | 0.214 | 0.069 | 0.068 | 1.281 | 482.72 | 0.618 | 0.697 | 0.476 | 0.144 | 0.903 | 0 |
| 17 | 2 | 1.5 | 0.2 | 0.866 | 0.826 | 1.45 | 200.775 | 0.544 | 0.546 | 0.538 | 0.046 | 0.096 | 0.081 | 0.061 | 1.283 | 578.955 | 0.671 | 0.698 | 0.474 | 0.147 | 0.714 | 0 |
| 18 | 2 | 1.5 | 0.1 | 0.867 | 0.821 | 1.516 | 214.8 | 0.545 | 0.547 | 0.528 | 0.052 | 0.047 | 0.119 | 0.049 | 1.28 | 619.979 | 0.685 | 0.697 | 0.468 | 0.153 | 0.441 | 0 |
| 19 | 1 | 0.6 | 0.5 | 0.805 | 0.127 | 0.516 | 212.501 | 0.544 | 0.544 | 0.544 | 0.028 | 0.234 | 0.04 | 0.232 | 1.279 | 596.584 | 0.626 | 0.464 | 0.712 | 0.119 | 0.968 | 0 |
| 20 | 1 | 0.6 | 0.2 | 0.804 | 0.121 | 0.533 | 233.331 | 0.545 | 0.547 | 0.537 | 0.031 | 0.137 | 0.073 | 0.223 | 1.281 | 516.18 | 0.544 | 0.465 | 0.711 | 0.123 | 0.941 | 0 |
| 21 | 1 | 0.6 | 0.1 | 0.804 | 0.104 | 0.555 | 256.692 | 0.546 | 0.548 | 0.529 | 0.033 | 0.056 | 0.123 | 0.215 | 1.284 | 494.786 | 0.511 | 0.469 | 0.709 | 0.127 | 0.803 | 0 |
| 22 | 1 | 1 | 0.5 | 0.76 | 0.138 | 0.862 | 369.632 | 0.547 | 0.547 | 0.547 | 0.049 | 0.408 | 0.019 | -0.28 | 1.281 | 766.204 | 0.591 | 0.592 | 0.589 | 0.181 | 0.995 | 0 |
| 23 | 1 | 1 | 0.2 | 0.76 | 0.128 | 0.915 | 404.779 | 0.547 | 0.548 | 0.543 | 0.054 | 0.252 | 0.037 | -0.286 | 1.283 | 800.313 | 0.593 | 0.593 | 0.59 | 0.186 | 0.96 | 0 |
| 24 | 1 | 1 | 0.1 | 0.761 | 0.131 | 0.955 | 415.585 | 0.548 | 0.549 | 0.538 | 0.058 | 0.114 | 0.061 | -0.3 | 1.282 | 813.662 | 0.592 | 0.592 | 0.585 | 0.185 | 0.805 | 0 |
| 25 | 1 | 1.5 | 0.5 | 0.711 | 0.563 | 1.371 | 306.41 | 0.546 | 0.546 | 0.546 | 0.056 | 0.353 | 0.038 | -0.92 | 1.282 | 957.497 | 0.591 | 0.661 | 0.483 | 0.195 | 0.967 | 0 |
| 26 | 1 | 1.5 | 0.2 | 0.711 | 0.586 | 1.444 | 313.02 | 0.546 | 0.548 | 0.541 | 0.062 | 0.216 | 0.048 | -0.93 | 1.28 | 1102.665 | 0.636 | 0.661 | 0.48 | 0.193 | 0.808 | 0 |
| 27 | 1 | 1.5 | 0.1 | 0.711 | 0.58 | 1.488 | 320.775 | 0.546 | 0.548 | 0.535 | 0.068 | 0.089 | 0.076 | -0.96 | 1.281 | 1156.022 | 0.649 | 0.661 | 0.478 | 0.195 | 0.541 | 0 |

Note: TG-ROC boundaries for strong tests are unavailable

Table D legend:

M1, sd1: Mean difference and standard deviation ratio between the two distributions

AUC: Area under the Curve

Range: lower and upper decision threshold of the trichotomization methods

Size: Mean number of individuals with test scores for all test scores, and inside the interval and range respectively

CCR: correct classification rate

Se, sp: Sensitivity and specificity of the test scores inside the interval and range respectively

P(t): proportion of simulations that showed significant mean differences between the group with and without the targeted condition

P.NA: proportion of simulations that did not allow for determination of the Uncertain Interval or Intermediate Range

Note: These results are equal to those of table 6, except the non-availability of TG-ROC’s Ranges for the stronger tests (column P.Na).
